# A Hybrid Deep Learning Model for Predicting Protein Hydroxylation Sites

**DOI:** 10.3390/ijms19092817

**Published:** 2018-09-18

**Authors:** Haixia Long, Bo Liao, Xingyu Xu, Jialiang Yang

**Affiliations:** 1 Department of Information Science and Technology, Hainan Normal University, Haikou 571158, China; myresearch_hainnu@163.com; 2 Department of Mathematics and Statistics, Hainan Normal University, Haikou 571158, China; 3 College of Life Sciences, Zhejiang Sci-Tech University, Hangzhou 310018, China; xingyuxu821@163.com; 4 Department of Genetics and Genomic Sciences, Icahn School of Medicine at Mount Sinai, New York, NY 10029, USA

**Keywords:** protein post-translational modification (PTM), hydroxylation sites, convolutional neural network (CNN), long short-term memory network (LSTM), iHyd-PseAAC, iHyd-PseCp

## Abstract

Protein hydroxylation is one type of post-translational modifications (PTMs) playing critical roles in human diseases. It is known that protein sequence contains many uncharacterized residues of proline and lysine. The question that needs to be answered is: which residue can be hydroxylated, and which one cannot. The answer will not only help understand the mechanism of hydroxylation but can also benefit the development of new drugs. In this paper, we proposed a novel approach for predicting hydroxylation using a hybrid deep learning model integrating the convolutional neural network (CNN) and long short-term memory network (LSTM). We employed a pseudo amino acid composition (PseAAC) method to construct valid benchmark datasets based on a sliding window strategy and used the position-specific scoring matrix (PSSM) to represent samples as inputs to the deep learning model. In addition, we compared our method with popular predictors including CNN, iHyd-PseAAC, and iHyd-PseCp. The results for 5-fold cross-validations all demonstrated that our method significantly outperforms the other methods in prediction accuracy.

## 1. Introduction

As a type of post-translational modification, hydroxylation converts a CH group into a COH group in a protein [1]. Protein hydroxylation usually happens in proline and lysine residues, which are called hydroxyproline and hydroxylysine, respectively. Hydroxyproline plays critical roles in collagen stabilization [2] and the development of a few cancers, such as stomach cancer [3] and lung cancer [4], while hydroxylysine contributes to fibrillogenesis, cross-linking, and matrix mineralization [5]. Consequently, predicting hydroxyproline and hydroxylysine sites in proteins may provide useful information for both biomedical research and drug development.

Nowadays, mass spectrometry is the most commonly used experiment in identifying hydroxylation residues [1,6], which is known to be time and labor intensive. With the development of high-throughput sequencing techniques, more and more protein sequences have been sequenced and stored, which presents an unprecedented opportunity as well as a big challenge for computational methods to predict hydroxylation residues in proteins. As a result, there are a few attempts in predicting hydroxylation residues using machine learning-based methods. For example, in 2009, Yang et al. [7] classified collagen hydroxyproline sites by developing two support vector machines with the identity kernel function and bio-kernel function respectively. In 2010, Hu et al. predicted hydroxyproline and hydroxylysine sites using a novel protein sequence-based method [8]. To predict carbamylated lysine sites, Huang et al. [9] presented a two-stage feature selection, followed by a one-class k-nearest neighbor classification method. In addition, Xu et al. [10] proposed a predictor called iHyd-PseAAC to predict protein hydroxylation sites. Qiu et al. [11] presented an ensemble classifier by integrating the random forest algorithm and other classifiers fusing different pseudo components. However, despite the improvement in the field, the prediction accuracy is still far from satisfactory. Deep learning-based methods have been proven to be effective in many bioinformatics problems, which might be a promising direction for further research in the area.

In this study, we develop a new predictor for identifying hydroxyproline and hydroxylysine in proteins by a hybrid deep learning model convolutional neural network (CNN) [12,13] and long short-term memory network (LSTM), one of the recurrent neural networks (RNNs) [13,14]. CNN uses the convolution layer to capture higher-level abstraction features of amino acid, and the recurrent layer of LSTM captures long-term dependencies between amino acids to improve the predictor quality. 

## 2. Results and Discussions

**Figure 1:**
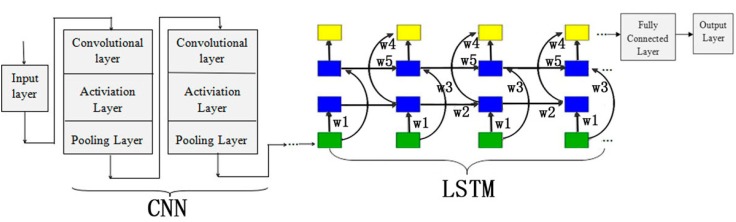
Architecture of CNN+LSTM for predicting phosphorylation sites. CNN: convolutional neural network; LSTM: long short-term memory network. Gray squares represent the layers of CNN; Green squares represent the first layers of LSTM; Blue squares represent the hidden layers of LSTM; Yellow squares represent the output layers of LSTM.

In our experiments, we used a scalable deep learning framework MXNET on CPU to implement our CNN+LSTM and CNN models, and our framework is illustrated in Figure 1. MXNET is a flexible and efficient library for deep learning. In order to test the performance of predicting hydroxylation predictor, CNN+LSTM compared with other classification algorithms, that is, CNN, iHyd-PseAAC [10], and iHyd-PseCp [11] implement on R programming language. 

There are three kinds of cross-validation methods: the n-fold cross-validation, the jackknife cross-validation, and the independent data test [15]. Among the three tests, the jackknife test has been widely used in bioinformatics because it could produce a unique outcome [16,17,18,19,20]. However, it is time- and source-consuming. Thus, in this paper, we used the 5-fold cross-validation to evaluate the proposed models.

We summarized in Table 1 the sensitivity (*Sn*), specificity (*Sp*), accuracy (*Acc*), and the Matthews correlation coefficient (*Mcc*) of the 5-fold cross-validation for 4 methods including CNN+LSTM, CNN, iHyd-PseCp, and iHyd-PseAAC on Dataset 1. Clearly, CNN+LSTM outperformed other methods in almost all criteria. The reason is that deep learning models with convolution layer capture regulatory motifs, while the recurrent layer captures long-term dependencies among the motifs, which improves predicting performance. Among all the predictors, the performance of iHyd-PseAAC is the worst, while CNN and iHyd-PseCp have comparative results. In iHyd-PseCp, the authors adopted the Random Forest algorithm. In addition, we summarized comparison results of the 4 methods on Dataset 2, Dataset 3, Dataset 4, Dataset 5, and Dataset 6 in Table 2, Table 3, Table 4, Table 5 and Table 6 respectively. As can be seen, the results are quite similar to that of Dataset 1. 

**Figure 2:**
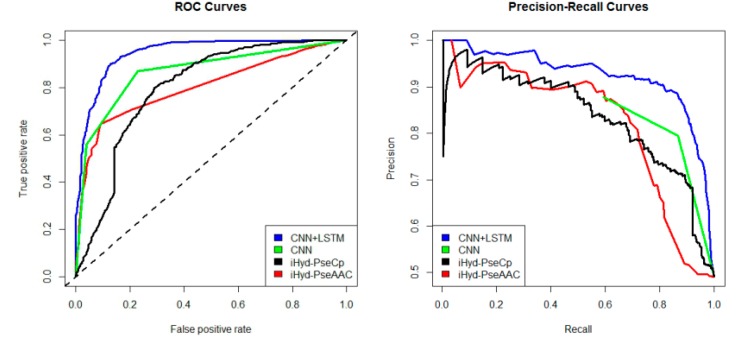
The receiver operating characteristic curve (ROC) and Precision-Recall curves to show the performance of CNN+LSTM, CNN, iHyd-PseCp and iHyd-PseAAC for the peptide samples with the center residue being proline on Dataset 1.

**Figure 3:**
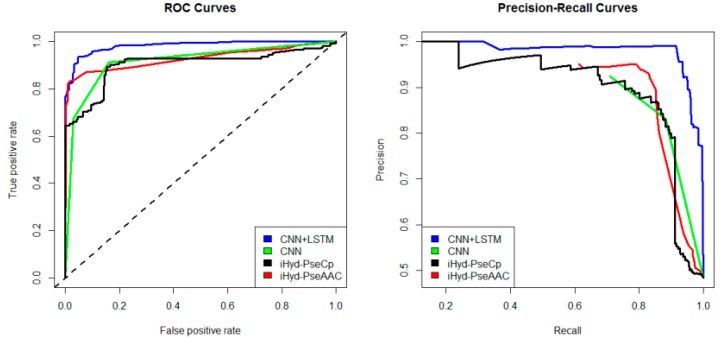
The ROC and Precision-Recall curves to show the performance of CNN+LSTM, CNN, iHyd-PseCp and iHyd-PseAAC for the peptide samples with the center residue being proline on Dataset 2.

**Figure 4:**
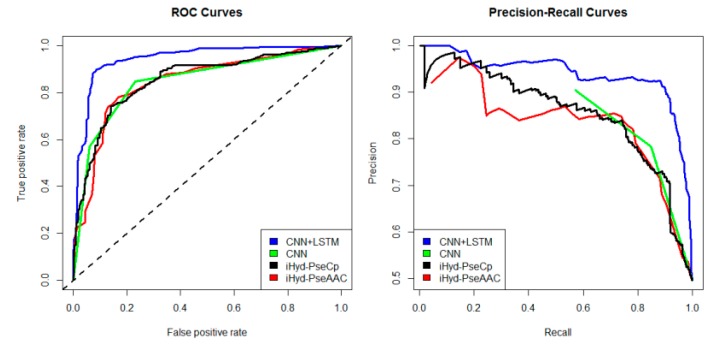
The ROC and Precision-Recall curves to show the performance of CNN+LSTM, CNN, iHyd-PseCp and iHyd-PseAAC for the peptide samples with the center residue being proline on Dataset 3.

**Figure 5:**
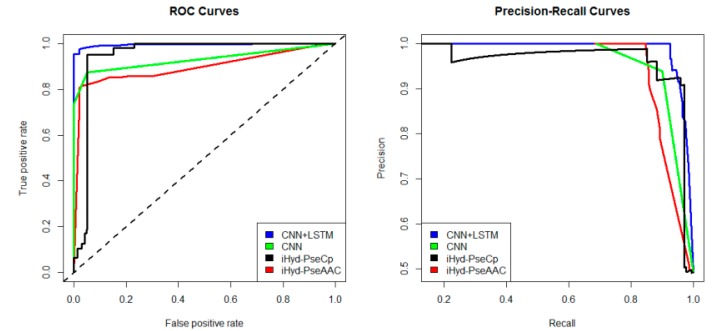
The ROC and Precision-Recall curves to show the performance of CNN+LSTM, CNN, iHyd-PseCp and iHyd-PseAAC for the peptide samples with the center residue being proline on Dataset 4.

**Figure 6:**
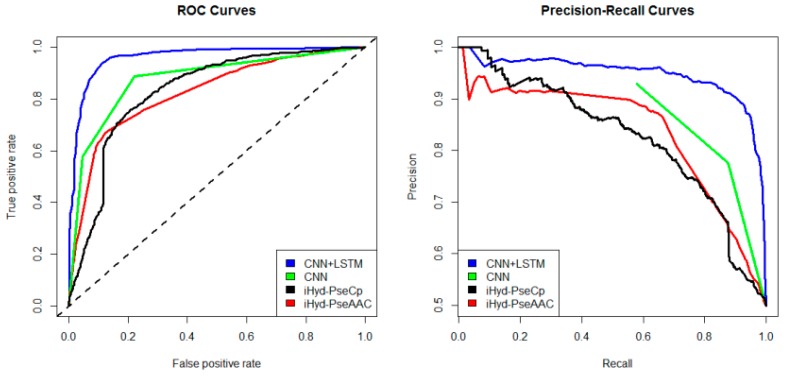
The ROC and Precision-Recall curves to show the performance of CNN+LSTM, CNN, iHyd-PseCp and iHyd-PseAAC for the peptide samples with the center residue being proline on Dataset 5.

**Figure 7:**
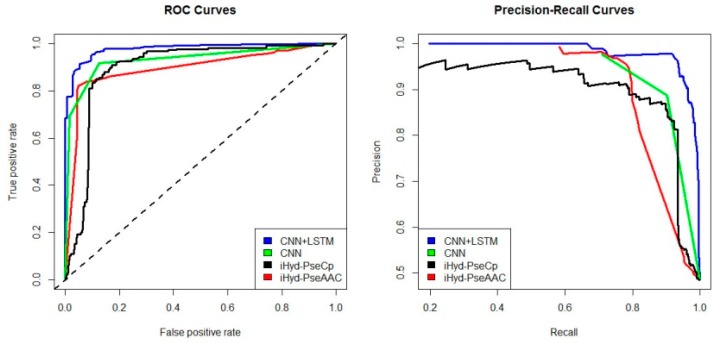
The ROC and Precision-Recall curves to show the performance of CNN+LSTM, CNN, iHyd-PseCp and iHyd-PseAAC for the peptide samples with the center residue being proline on Dataset 6.

In addition, we plotted in Figure 2 the average receiver operating characteristic curve (ROC) and precision-recall (PR) curves of the 4 tested methods for the peptide samples of the center residue being proline and lysine on Dataset 1. We also calculated the area under ROC curve (AUC) for each method respectively, which yields AUC_CNN+LSTM_ = 0.96, AUC_CNN_ = 0.83, AUC_iHyd-PseCp_ = 0.81, and AUC_iHyd-PseAAC_ = 0.70. Our method achieves the highest AUC 0.96, suggesting that it is better than other methods in prediction accuracy. Similarly, we also plotted the average ROC and precision-recall (PR) curves on datasets 2–6 in Figure 3, Figure 4, Figure 5, Figure 6 and Figure 7 respectively. Specifically, the AUC values in Figure 3 are AUC_CNN+LSTM_ = 0.98, AUC_CNN_ = 0.91, AUC_iHyd-PseCp_ = 0.89, and AUC_iHyd-PseAAC_ = 0.86, respectively. The AUC values in Figure 4 are AUC_CNN+LSTM_ = 0.93, AUC_CNN_ = 0.85, AUC_iHyd-PseCp_ = 0.86, and AUC_iHyd-PseAAC_ = 0.84, respectively. The AUC values in Figure 5 are AUC_CNN+LSTM_ = 0.99, AUC_CNN_ = 0.91, AUC_iHyd-PseCp_ = 0.96, and AUC_iHyd-PseAAC_ = 0.89, respectively. In Figure 6, AUC_CNN+LSTM_ = 0.96, AUC_CNN_ = 0.84, AUC_iHyd-PseCp_ = 0.83, and AUC_iHyd-PseAAC_ = 0.80. In Figure 7, AUC_CNN+LSTM_ = 0.97, AUC_CNN_ = 0.85, AUC_iHyd-PseCp_ = 0.84, and AUC_iHyd-PseAAC_ = 0.81. CNN+LSTM achieved remarkably greater AUCs than other methods in all datasets, further demonstrating the excellent performance of our model.

Finally, we computed the *p*-value assessing the significance of the difference between two AUCs (http://vassarstats.net/roc_comp.html). For Figure 2, except for CNN and iHyd-PseCp (with *p*-value 0.18309), all *p*-values between AUCs of other methods are less than 0.000001. The results indicate that our method is significantly better than the compared methods. Similar results were observed for all datasets. For example, in Figure 3 the *p*-values between CNN+LSTM and CNN, between CNN+LSTM and iHyd-PseCp, and between CNN+LSTM and iHyd-PseAAC are 0.001161, 0.000099, and 0.000002, respectively. The corresponding values for Figure 4 are 0.000007, 0.000058, <0.000001, respectively. In summary, CNN+LSTM is significantly better than CNN, iHyd-PseCp, and iHyd-PseAAC in predicting AUCs across all 6 datasets. 

## 3. Methods

### 3.1. Benchmark Dataset

The benchmark dataset consists of 164 hydroxyproline proteins and 33 hydroxylysine proteins, which were also used by Xu et al. [10] and Qiu et al. [11]. Because the length of proteins is different and the position of the hydroxylation sites is not the same, peptide sample presentation [21,22,23] proposed by Chou were adopted to obtain the same length of samples. A peptide sample *α* can be expressed as following:

(1) $$\;Q_{\varphi }\left(\alpha \right)=R_{-\varphi }R_{-\left(\varphi -1\right)}\cdots R_{-2}R_{-1}\;\alpha \;R_{+1}R_{+2}\cdots R_{+\left(\varphi -1\right)}R_{+\varphi }\;$$

where the symbol *α* denotes the single amino acid code P or K, the subscript φ is an integer, $R_{-\varphi }\;$represents the φ-th downstream amino acid residue from the center, and $R_{+\varphi }$ represents the φ-th upstream amino acid residue. Peptides Q can be further classified as:

(2) $$\;Q_{\varphi }\left(\alpha \right)\epsilon \{\begin{array}{c} Q_{\varphi }^{+}\left(\alpha \right),\;if\;its\;center\;is\;a\;hydroxylation\;site\\ Q_{\varphi }^{-}\left(\alpha \right),\;otherwise\; \end{array}\;$$

**Figure 8:**
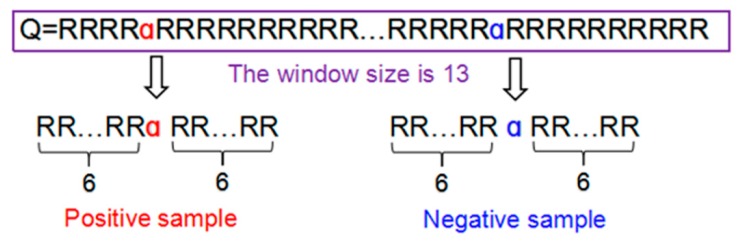
The process of formulating the positive and negative peptide samples.

When φ=6, each of the samples extracted from proteins for this study is a 2φ + 1 = 13  tuple peptide. If the upstream or downstream residues in a peptide sample were 3 ≤ φ ≤ 6, the lacking residues were filled with the dummy code @. Figure 8 illustrated the process of formulating the positive and negative peptide samples.

To test the performance of the deep learning classifier proposed in the manuscript, we used the following six datasets to train and test the model shown in Table 7. The first four datasets were downloaded from iHyd-PseAAC [10] (http://app.aporc.org/ihyd-pseaac/), and the length of each sample is 13 (φ = 6). The last two datasets were downloaded from iHyd-PseCp [11] (http://www.jci-bioinfo.cn/iHyd-PseCp), and the length of each sample is 21 (φ = 10). 

From Table 7, we can see that the number of positive and negative samples is not imbalanced. The number of negative samples is far greater than that of the positive samples. A predictor can easily overfit the data to achieve higher accuracy because most samples belong to the negative class. To address this problem, we used the bootstrapping method proposed by Yan et al. [24], which is described as follows: First, we split the imbalanced training data into negative and positive samples. Let *n* be the number of negative samples, and *p* be the number of positive samples. For each bootstrapping iteration, we selected the same number of positive and negative samples (*Sp*), then train the prediction model on the balanced data. In order to use all the negative samples, we divided the *n* negative samples into *N* bins such that each bin has *Sp* (*N* = [*n*/*Sp*]). Finally, we generate one training predictor through *N* number of bootstrap iterations. The details about the method and parameters setting can be obtained from reference [24]. 

### 3.2. Feature Extraction

A statistical method for predicting the hydroxylation sites of peptides in proteins is necessary. According to [21], the general form of PseAAC (pseudo amino acid composition) for a protein or peptide, *Q*, can be formulated as:

(3) $$\;Q={\left[\Psi_{1}\;\Psi_{2}\;\cdots \;\Psi_{\mu }\;\cdots \;\Psi_{\Omega }\right]}^{T}\;$$

where *Ω* is the vector’s dimension and it is an integer, *T* is the transpose operator. In Equation (3), *Ω* value and each component must be able to extract the essential feature from peptide samples, so position-specific scoring matrix (PSSM) is adopted, which is shown in Equation (4).

(4) $$\;Q_{PSSM}^{\left(0\right)}=\left[\begin{array}{c} m_{1,1}^{\left(0\right)}\;m_{1,2}^{\left(0\right)}\;\cdots \;m_{1,20}^{\left(0\right)}\\ \;m_{2,1}^{\left(0\right)}\;m_{2,2}^{\left(0\right)}\;\cdots \;m_{2,20}^{\left(0\right)}\;\\ \;\vdots \;\vdots \;\vdots \;\vdots \;\\ m_{L,1}^{\left(0\right)}\;m_{L,2}^{\left(0\right)}\;\cdots \;m_{L,20}^{\left(0\right)} \end{array}\right]\;$$

where subscript *L* is the length of a peptide sample, *L* = 13 or *L* = 21. Subscript values 1, 2 ⋯, 20 represent the 20 amino acid types based on the alphabetical order. $m_{i,j}^{\left(0\right)}$ denotes the original score of amino acid residue in the *i*-th (i=1, 2, ⋯,  L) sequential position that is changed to amino acid type (j=1, 2,⋯, 20) in the process of evolution. All the values in PSSM can be generated by using PSI-BLAST [25] according to the following steps. Step 1: Select the UniProtKB/Swiss-Prot databases. Step 2: Enter the peptide samples. Step 3: Set the *E*-value cut-off is 0.001. Then you can submit to obtain the PSSM. Finally, using standard sigmoid function can make every element in (4) within the range of [0, 1], which is shown in Equation (5).

(5) $$\;Q_{PSSM}^{\left(0\right)}=\left[\begin{array}{c} m_{1,1}^{\left(0\right)}\;m_{1,2}^{\left(0\right)}\;\cdots m_{1,20}^{\left(0\right)}\\ \;m_{2,1}^{\left(0\right)}\;m_{2,2}^{\left(0\right)}\;\cdots \;m_{2,20}^{\left(0\right)}\;\\ \;\vdots \;\vdots \;\vdots \;\vdots \;\\ m_{L,1}^{\left(0\right)}\;m_{L,2}^{\left(0\right)}\;\cdots \;m_{L,20}^{\left(0\right)} \end{array}\right]\;$$

where

(6) $$\;m_{1,j}^{\left(1\right)}=\frac{1}{1+e^{-m_{i,j}^{\left(0\right)}}}\;$$

### 3.3. A Hybrid Deep Learning Model

A convolutional neural network (CNN) is a deep learning model, which core layer is convolution layer. The convolution layer consists of a set of filters. Each filter is convolved across dimensions of input data, producing a multidimensional feature map. The CNN will learn filters that activate when they see some specific type of feature at some spatial position in input. The key architectural characteristics are local connectivity and shared weights.

Another deep learning model is recurrent neural network (RNN). Unlike feed forward neural networks, RNNs can use their internal state (memory) to process sequences of inputs. The architecture of convolutional and recurrent deep learning neural network for predicting hydroxylation sites is shown in Figure 2. The first layer is the input layer, which takes the PSSM matrix of each sample as inputs. The second layer is the convolution layer, which contains 320/1024 kernels. Its window size is 26/30 and step size is 1. Specifically, the convolution layer exacts and learns the input features. The pooling layer keeps the main features and reduces the number of parameters and the calculation of the next layer. Its window size is 13/15 and step size is 13/15. The bi-directional long short term memory layer contains 320/512 forward and 320/512 backward LSTM neurons, which can capture previous and future features. The fully connected layer consists of 925 neurons, which acts as classifier by using the sigmoid function and outputting the probability of classes. The last layer is the output layer, which outputs the final labels.

In addition, the regularization parameters are set as follows: the dropout proportion of outputs are randomly set to 0; the dropout proportion of Layer 2, Layer 3 and all other layers are 20%, 50% and 0%, respectively.

### 3.4. A Set of Four Metrics for Measuring Prediction Quality

This study measures prediction quality using the Chou set of four metrics [21,22,23] to predict signal peptides. They are sensitivity (*Sn*), specificity (*Sp*), accuracy (*Acc*), and the Matthews correlation coefficient (*Mcc*), respectively.

(7) $$\;Sn=1-\frac{N_{-}^{+}}{N^{+}}\;\;Sp=1-\frac{N_{+}^{-}}{N^{-}}\;\;Acc=1-\frac{N_{-}^{+}+N_{+}^{-}}{N^{+}+N^{-}}\;\;Mcc=\frac{1-\left(\frac{N_{-}^{+}+N_{+}^{-}}{N^{+}+N^{-}}\right)}{\sqrt{\left(1+\frac{N_{+}^{-}-N_{-}^{+}}{N^{+}}\right)\left(1+\frac{N_{-}^{+}-N_{+}^{-}}{N^{-}}\right)}}\;$$

where $N^{+}$ is the number of the positive samples and $N^{-}$ is the number of the negative samples, $N_{-}^{+}$ is the number of positive samples incorrectly predicted as negative samples and $N_{+}^{-}$ is the number of negative samples incorrectly predicted as positive samples. 

### 3.5. Receiver Operating Characteristics (ROC) and Precision-Recall (PR) Curve to Evaluate the Classification Quality

To evaluate the performance of the predictor, we not only used the above four metrics, but also used graphical approach. Receiver operating characteristic curve (ROC) [26] and precision-recall curve (PR) are utilized to show all the results from intuitive comparison. 

For a binary classification, if a sample is positive and it is predicted positive sample, then it is true positive (TP), if a sample is negative and it is predicted positive sample, then it is false positive (FP), if a sample is negative and it is predicted negative sample, then it is true negative (TN), if a sample is positive and it is predicted negative sample, then it is false negative (FN). ROC curve can be plotted by the true positive rate (TPR) against the false positive rate (FPR) and PR curve can be plotted by the precision against the recall at various threshold settings. The area under the ROC curve is called AUC. The greater the AUC value, the better the predictor will be. 

(8)  TPR=TP/(TP+FN) FPR=FP/(FP+TN) precision=TP/(TP+FP) recall=TP/(TP+FN) 

## 4. Conclusions

In this study, we have proposed a hybrid deep learning model CNN+LSTM for predicting hydroxylation sites. The comparison with other popular methods including iHid-PseACC and IHyd-PseCp demonstrates that our method is superior in prediction accuracy. However, our model has a few limitations. Firstly, just like other deep leaning models, the proposed model is slower than other classification methods, such as random forest, the support vector machine and the k-nearest neighbor method. The structure complexity of the model and the time complexity of the forward–backward algorithm and gradient descent algorithm at least contribute partially to the inefficiency of our algorithm in the training step. Secondly, our model has a lot of parameters, such as the number of layers, the number of kernels, the number of neurons in each layer, the weight of each neuron, and so on. Tuning the optimal parameters is time-consuming and error-prone. 

In the future, to improve the efficiency of the new prediction method, a userfriendly and publicly accessible web server is often established [27,28,29,30,31]. Hence, we will also make efforts to provide a web server for the proposed method in our future studies that will be useful to the vast majority of experimental scientists. In addition, we will improve the architectures of the deep learning model and seek the optimal parameters.

## Figures and Tables

**Table 1:** A comparison of predictors for identifying hydroxyproline sites on Dataset 1.

Method	*Sn*	*Sp*	*Acc*	*Mcc*
CNN+LSTM	94.52	97.43	90.68	0.91
CNN	87.49	90.43	94.16	0.86
iHyd-PseCp	86.23	90.16	93.48	0.85
iHyd-PseAAC	79.58	78.42	79.69	0.60

*Sn*: sensitivity; *Sp*: specificity; *Acc*: accuracy; *Mcc*: Matthews correlation coefficient.

**Table 2:** A comparison of predictors for identifying hydroxylysine sites on Dataset 2.

Method	*Sn*	*Sp*	*Acc*	*Mcc*
CNN+LSTM	97.30	99.84	93.27	0.94
CNN	90.43	91.38	90.59	0.90
iHyd-PseCp	90.84	93.23	90.63	0.89
iHyd-PseAAC	86.72	82.53	85.09	0.68

**Table 3:** A comparison of predictors for identifying hydroxyproline sites on Dataset 3.

Method	*Sn*	*Sp*	*Acc*	*Mcc*
CNN+LSTM	92.24	95.72	89.15	0.90
CNN	83.27	86.93	83.60	0.84
iHyd-PseCp	88.62	89.42	85.64	0.85
iHyd-PseAAC	73.54	86.71	75.32	0.68

**Table 4:** A comparison of predictors for identifying hydroxylysine sites on Dataset 4.

Method	*Sn*	*Sp*	*Acc*	*Mcc*
CNN+LSTM	98.84	97.66	96.91	0.97
CNN	89.15	88.75	84.76	0.85
iHyd-PseCp	92.05	91.82	90.53	0.89
iHyd-PseAAC	85.21	84.90	80.38	0.71

**Table 5:** A comparison of predictors for identifying hydroxyproline sites on Dataset 5.

Method	*Sn*	*Sp*	*Acc*	*Mcc*
CNN+LSTM	92.06	98.39	96.55	0.91
CNN	87.47	99.38	97.29	0.90
iHyd-PseCp	86.35	99.12	96.58	0.89
iHyd-PseAAC	80.66	80.54	80.57	0.51

**Table 6:** A comparison of predictors for identifying hydroxylysine sites on Dataset 6.

Method	*Sn*	*Sp*	*Acc*	*Mcc*
CNN+LSTM	94.75	98.53	97.19	0.89
CNN	89.94	99.27	97.57	0.88
iHyd-PseCp	78.77	99.80	97.08	0.86
iHyd-PseAAC	87.85	83.01	83.56	0.50

**Table 7:** Positive and negative peptide samples of proline and lysine site.

Samples	Window Size = 13				Window Size = 21	
	α = Proline	α = Lysine	α = Proline	α = Lysine	α = Proline	α = Lysine
Positive samples	636	107	306	44	851	142
Negative samples	2699	836	1035	528	3505	980
Total samples	3335	943	1341	572	4356	1122
